# The development of bronchoscopy in China: a national cross-sectional study

**DOI:** 10.7150/jca.47183

**Published:** 2020-07-20

**Authors:** Dongchen Shi, Fuqi Li, Kaicheng Wang, Chen Kong, Haidong Huang, Qiang Li, Faguang Jin, Chengping Hu, Changhui Wang, Hui Shi, Zhenli Hu, Yuchao Dong, Yunye Ning, Kosmas Tsakiridis, Konstantinos Sapalidis, Christoforos Kosmidis, Anastasios Vagionas, Wolfgang Hohenforst-Schmidt, Lutz Freitag, J Francis Turner, Konstantinos Drevelegas, Eleni-Isidora Perdikouri, Tomi Kovacevic, Tatjana Sarcev, Bojan Zaric, Savas Petanidis, Sofia Baka, Ioannis Boukovinas, Stylianos Kakolyris, Paul Zarogoulidis, Chong Bai

**Affiliations:** 1 Department of Respiratory and Critical Care Medicine, Changhai Hospital, The Second Military Medical University, Shanghai, China.; 2 Department of Respiratory, Oriental Hospital, Tongji University, Shanghai, China.; 3 Department of Respiratory, Tangdu Hospital, The Fourth Military Medical University, Xian, China.; 4 Department of Respiratory, Xiangya Hospital, Central South University, Changsha, China.; 5 Department of Respiratory, Shanghai Tenth People's Hospital, Tongji University, Shanghai, China.; 6 Thoracic Surgery Department, ``Interbalkan`` European Medical Center, Thessaloniki, Greece; 7 3rd Department of Surgery, ``AHEPA`` University Hospital, Aristotle University of Thessaloniki, Medical School, Thessaloniki, Greece; 8 Oncology Department, (NHS) General Hospital of Kavala, Kavala, Greece; 9 Sana Clinic Group Franken, Department of Cardiology / Pulmonology / Intensive Care / Nephrology, "Hof" Clinics, University of Erlangen, Hof, Germany.; 10 Department of Pulmonology, University Hospital Zurich, Zurich, Switzerland.; 11 University of Tennessee Graduate School of Medicine, Department of Medicine, Knoxville, TN, USA.; 12 Radiology Department, ``G. Papageorgiou`` University Hospital, Aristotle University of Thessaloniki, Thessaloniki, Greece; 13 Oncology Department, General Hospital of Volos, Volos, Greece; 14 Institute for Pulmonary Diseases of Vojvodina, Faculty of Medicine, University of Novi Sad, Serbia; 15 Department of Pulmonology, I.M. Sechenov First Moscow State Medical University, Moscow, Russian Federation; 16 Oncology Department, ``Interbalkan`` European Medical Center, Thessaloniki, Greece; 17 Oncology Department, ``Bioclinic`` Private Hospital, Thessaloniki, Greece; 18 Oncology Department, University General Hospital of Alexandroupolis, Democritus University of Thrace, Alexandroupolis, Greece

**Keywords:** bronchoscopy, education, Respiratory diseases

## Abstract

**Objective:** To investigate the development of bronchoscopy in China and compare it with its application in the early 21st century.

**Methods:** The data collection was based on questionnaires. Three hundred and nineteen hospitals, which distributed across 30 provinces and 130 cities, were included in the study. Data about the application of bronchoscopy in Shanghai and Hunan province in the early 21st century are also involved for comparison.

**Results:** The median period of performing diagnostic and therapeutic bronchoscopy was 19.7±11.0 and 7.4±7.0 years, respectively. On average, about 155.2 cases and 28.4 cases received diagnostic and therapeutic bronchoscopy in each hospital per month. The average area and number of the examination room was 122.7m^2^ and 2.2m^2^, respectively. More examination items were performed in specialty hospitals than those in general hospitals (P<0.05) and specialty hospitals owned more rooms exclusively for bronchoscopy (P<0.05), while no difference of the number of allocated doctors was found (P>0.05). On the other side, the whole amount of diagnosis and therapeutic items in teaching hospitals was slightly higher than that in non-teaching hospitals (P<0.01). Comparison of diagnosis and therapeutic endoscopy in Shanghai and Hunan province shows that the number of flexible bronchoscopy increased by 5.8 times in Shanghai from 2002 to 2017, while that increased by 3.4 times in Hunan province from 2005 to 2017. Furthermore, the average number of allocated doctors increased by 0.85 times in Shanghai, which was more rapidly compared with that of Hunan province (0.66 times) (P<0.05). Besides, the development rate of the diagnosis and therapeutic projects in Shanghai was significantly higher than that in Hunan province (P<0.05).

**Conclusion:** All different classes of hospitals in China are capable of carrying out conventional bronchoscopy diagnosis and therapeutic projects, and newly developed bronchoscopy technology has gradually spread in high-level hospitals since 21st century. The higher class the hospital was, the earlier bronchoscopy was performed. Respiratory endoscopy in China has developed rapidly since the early 21st century and the construction of respiratory endoscopy center and the personnel training are on the right track, but it is also faced with inadequate equipment, unbalanced regional development and insufficient personnel allocation.

## Introduction

Bronchoscopy was an endoscopic technique of visualizing the inside of the airways for diagnostic and therapeutic purposes, including investigation of lesions in the bronchi, fine needle aspiration of the tumor, hemostasis and stent implantation for bronchial stenosis, which was pivotal for amelioration of local symptoms and subsequent treatment after confirmed diagnosis 1. Novel endoscopic techniques are being used nationwide in China such as the radial endobronchial ultrasound, convex probe (EBUS), electromagnetic navigation and the archimedes virtual bronchoscopy system. Additionally, where necessary the transthoracic ultrasound convex probe is being used in order to biopsy superficial lesions of the thorax 2-9. There already have been international reports on the diagnosis and treatment of respiratory endoscopy in the United States, Britain, Japan and other countries from 1986 to 2010 10-15. In 2002, Bai C. et al. 16 made a statistical analysis about the clinical application of the bronchoscopy in Shanghai, which was initial internationally recorded investigation report on the application of bronchoscopy in China. In 2009, Nie X. et al. 17 reported clinical practice of bronchoscopy in some limited areas of China on Chest. Therefore, in order to make a comprehensive understanding of development of bronchoscopy in the recent 10 years in China, the study was performed with network questionnaires nationwide in 2017.

## Materials and Methods

### Object of the survey

The survey was conducted by network questionnaire. The items of questionnaire were determined according to interventional technology developed home and abroad. Then the network questionnaire was filled in with real-time data by the hospitals surveyed from June 2017 to August 2017. The survey was distributed across 30 provinces and 130 cities and all classes of hospitals were involved, which could represent the current situation of bronchoscopy in China during the survey period. A total of 319 hospitals were included in this survey, which were located across the whole country. Among these hospitals, there were 221 first-class (69.3%) and 38 second-class (11.9%) tertiary hospitals. Also, 56 first-class secondary hospitals (17.6%) and 4 second-class (1.2%) secondary hospitals were enrolled in this study. A 90% valid response (319 questionnaires out of 356) was obtained in final data. The effective rate of tertiary hospitals was 90.9% (259/285) and that of secondary hospitals was 84.5% (60/71). Moreover, those 319 hospitals were also divided into 305 general, 14 specialty, 113 teaching and 206 non-teaching hospitals. Among all the 319 valid questionnaires, the valid rates of specialty hospitals were 63.6% (14/22), general hospitals 91.3% (305/334), teaching hospitals 85.6% (113/132) and non-teaching hospitals 92.0% (206/224). Besides, we had also collected data on practice of flexible bronchoscopy in Shanghai collected in 2002 and 2017 and those in Hunan province collected in 2005 and 2017, in order to analyze the trend of development of bronchoscopy in different regions in China.

From June 2017 to August 2017, 14 tertiary and secondary hospitals and thoracic and pulmonary hospitals in Shanghai filled out the questionnaire online. The final valid questionnaire covered 11 hospitals, with an effective rate of 78.6%. From February to April 2002, 111 hospitals in Shanghai were surveyed and 52 questionnaires were collected, with a valid response rate of 46.8%.

### Research contents

The collection of data was based on questionnaires. The required information was as follows:

- the period of performance of diagnostic and therapeutic bronchoscopy;
- detailed items of diagnostic bronchoscopy: bronchoalveolar lavage (BAL), biopsy with forceps, transbronchial needle aspiration (TBNA), narrow band imaging (NBI), thoracoscopy, endobronchial ultrasound system with a guide sheath (EBUS-GS), percutaneous lung biopsy, virtual bronchoscopy (VB), electromagnetic navigation bronchoscopy (ENB), cryobiopsy and microwave examination;
- detailed items of therapeutic bronchoscopy: electrocautery, laser ablation, balloon dilation, argon plasma coagulation (APC), bronchoscopic lung volume reduction (BLVR), bronchoplasty, stent implantation, radioactive seeds implantation and microwave therapy;
- the number of diagnostic bronchoscopy, therapeutic bronchoscopy performed per month;
- the number of routine examination items performed per month: brush cytology, biopsy with forceps, TBNA, EBUS-TBNA;
- the number and area of the examination room for bronchoscopy;
- the number of equipment: electronic bronchoscope, fiberoptic bronchoscope, rigid bronchoscope, auto-fluorescence bronchoscope, EBUS, cryotherapy, electrocautery, APC, laser ablation, microwave therapy, flexible thoracoscope, rigid thoracoscope;
- the number of allocated doctors and anesthetists: number of doctors, number of doctors with doctoral degrees, number of doctors with master's degrees, number of anesthesiologists, number of anesthesiologists with doctoral degrees, number of anesthesiologists with master's degrees.

### Data management and quality assurance and statistical analysis

After completing the collection of online questionnaires, the data are screened uniformly to find out the technical errors in the filling process. Questionnaires that didn't conform to the standards and failed to fill in data were invalid and not included in the statistics. Categorical data were shown as percentages. All reported data from each hospital were gathered. Afterwards, data would be kept in a password-protected database at the Department of Pulmonary and Critical Care Medicine in Changhai Hospital, which would only be disclosed to authorized individuals. The accuracy of the data entry into the database would be confirmed by two administrators.

## Results

### Clinical practice of diagnosis and therapeutic bronchoscopy in China

#### The period of performance of diagnostic and therapeutic bronchoscopy

According to the survey, as of August 31, 2017, the average period of diagnostic and therapeutic bronchoscopy was 19.7±11.0 years and 7.4±7.0 years respectively. Diagnosis and therapeutic bronchoscopy was first developed in first-class tertiary hospitals. The larger the scale of hospitals, the earlier the diagnostic and therapeutic bronchoscopy would be developed (Table 1).

Shanghai Pulmonary Hospital, Shanghai Thoracic Hospital, Henan People's Hospital, Tianjin Thoracic Hospital and other hospitals have been initiated developing diagnostic bronchoscopy since 1971. The number of hospitals performing bronchoscopy in China has steadily increased. The development of therapeutic bronchoscopy began in the 1980s, showing a slow growth trend. Since 2000, the therapeutic bronchoscopy in China has entered a new period of rapid development.

The average period of bronchoscopy in specialty hospitals was 28.4 + 12.5 years, and the average time of treatment was 14.3 + 8.5 years. The average period of respiratory endoscopy in general hospitals was 19.3 + 10.7 years, and the average time of treatment was 7.1 + 6.7 years. The average time of respiratory endoscopy in teaching hospitals was 23.9 (+10.5) years, and the average time of treatment was 9.2 (+7.1) years. The average time of respiratory endoscopy in non-teaching hospitals was 17.4 (+10.5) years, and the average time of treatment was 6.3 (+6.4).

#### Items of diagnostic and therapeutic bronchoscopy

At present, there are dozens of clinical diagnostic and therapeutic techniques, which can be used to observe the tracheal tract and local lesions, tissue sampling, hemostasis, resection or reconstruction of the passage and so on. They play an irreplaceable role in clinical diagnosis and therapy. Through the statistics of the projects carried out by hospitals at all levels, we can understand the current clinical application of various diagnostic and therapeutic projects. Based on the intervention procedure we use sedation with propofol or general anesthesia with plastic tube intubation or rigid bronchoscope intubation (STORZ). Moreover; we use for all the patients receiving general anesthesia jet-ventilation respiratory model since this model keeps the CO_2_ low 18.

Among all the diagnostic items in 319 hospitals, BAL, biopsy, TBNA, percutaneous lung biopsy guided by CT and thoracoscopy were most frequently performed (more than 50%), which were available in 93.7%, 99.4%, 64.9%, 66.1% and 57.1% in hospitals. While other items, performed less than 50%, included EBUS-TBNA (34.5%), NBI (28.5%), EBUS-GS (27.6%), VB (16.0%) and ENB (4.4%). The detailed rates of diagnostic bronchoscopy in hospitals at all levels are shown in Table 2 and Table 3. The Chinese pathology techniques for sample assessment do not differ from the European guidelines.

Among all the therapeutic items in 319 hospitals, balloon dilatation (69.6%), electrocautery (65.5%), stent implantation (62.1%) and APC (52.7%) were most accessible to patients (more than 50%), while cryotherapy (47.0%), laser ablation (24.8%), microwave therapy (21.3%), bronchoplasty (18.8%). Other treatment projects that performed less than 25% included radioactive seeds implantation (17.9%) and LVR (17.2%). Details about various therapeutic technologies in tertiary and secondary hospitals were shown in Table 4.

Among the diagnostic projects performed by specialty hospitals, the launching rate of all items in teaching hospitals was higher than that in non-teaching hospitals except biopsy (P<0.05, Supplementary Table S1).

Among the therapeutic projects carried out by specialty hospitals, the developing rate of electrocoagulation, balloon dilation, microwave therapy and other therapeutic projects in specialty hospitals was higher than in general hospitals (P<0.05, Supplementary Table S2).

In the comparison of therapeutic projects carried out in teaching hospitals and non-teaching hospitals, The launching rate of therapeutic projects of the teaching hospital were much higher than that of non-teaching hospitals (P<0.05, Supplementary Table S3).

#### The number of cases receiving diagnostic and therapeutic bronchoscopy

In the 319 hospitals, 155.2 and 28.4 cases underwent diagnostic and therapeutic bronchoscopy in each hospital per months on average, respectively. Furthermore, 201 cases and 35.3 cases received diagnostic and therapeutic bronchoscopy in the first-class tertiary hospitals, respectively, while 79.4 cases and 18.1 cases were examined and treated by bronchoscopy in the second-class tertiary hospitals, respectively (Table 5).

Regarding area distributions, diagnostic bronchoscopy was mostly performed in Shanghai (436.4 cases), Sichuan province (336.2 cases), Shaanxi province (314.0 cases), Beijing (255.7 cases) and Hunan province (243.9 cases) per month, respectively. While therapeutic bronchoscopy was most frequently performed in Shanghai (77.4 cases), Shaanxi province (59.2 cases), Hunan province (49.8 cases), Beijing (45.6 cases) and Sichuan province (38.2 cases) per month respectively. Moreover, the areas with the highest number of bronchoscopy per million populations were Shanghai (21.23), Beijing (13.86) and Shanxi province (9.79).

If taking average number of cases of diagnosis and therapeutic bronchoscopy per hospital per month in each area as an indicator of the capacity of bronchoscopy for local residents. Therefore, Shanghai (21.23), Beijing (11.77) and Shaanxi (9.79) have more resources of respiratory endoscopic intervention per million resident populations.

### Allocated doctors and equipments

#### The area and number of respiratory endoscopic center

On average, the area and number of examination room for bronchoscopy was 122.7 m^2^ and 2.2 per hospital. Regarding tertiary hospitals, the average area of the examination room was 150.3 m^2^ and 99.7 m^2^ in first- and second-class hospitals, respectively. While there were 2.4 and 2.2 rooms for bronchoscopy in these two hospitals, respectively. Respiratory endoscopic center in tertiary hospitals tended to be broad and numerous (Supplementary Table S4).

The number of hospitals with the total area of respiratory endoscopic center less than 201 m^2^ accounted for 96.55% (308/319) of the total number of hospitals, and the area was mainly concentrated in the two intervals of 1~<26 m^2^ and 26~<51 m^2^. In the survey on the status of digestive endoscopy in China in 2015, the number of hospitals with the digestive endoscopic center covering less than 201 m^2^ accounted for 1.27% of all the hospitals, which were mainly concentrated in the two intervals of 26~<51m^2^ and 51~<101 m^2^.

#### The allocated equipment

Among 319 hospitals, there were 4.4 electronic bronchoscopes, 1.7 fiberoptic bronchoscopes, 0.9 APC, 0.8 Cryotherapy, 0.7 Electrocautery, 0.6 flexible thoracoscope, 0.3 rigid bronchoscope, 0.4 auto-fluorescence bronchoscope, 0.5 EBUS, 0.2 laser ablation, 0.2 microwave and 0.3 rigid thoracoscope in each hospital on average (Table 6).

Compared with other area in China, there were more flexible bronchoscopes (including electronic and fiberoptic bronchoscopes) on average per hospital in Shanghai (17.0), Beijing (12.7), Shanxi province (9.4), Tianjin (8.8) and Hubei province (7.9) (Table 6). Patient access to flexible bronchoscopes was calculated by the number per million populations. The areas with the highest ratio were Shanghai (0.7025), Beijing (0.5851) and Tianjin (0.5655). More details were shown in Supplementary Table S5.

#### The allocated doctors and anesthetists

On average, there were 7.4 doctors, 18.4% of which had doctoral degree, and 4.8 anesthetists, 9.8% of which had doctoral degree, specializing in bronchoscopy per hospital.

In detail, 8.3 doctors and 5.6 anesthetists were allocated for bronchoscopy in first-class tertiary hospitals; 6.5 doctors and 4.1 anesthetists majored in bronchoscopy in second-class tertiary hospitals. While 3.8 doctors and 2.4 anesthetists majored in bronchoscopy in first-class secondary hospitals; 3.5 doctors and 3.8 anesthetists majored in bronchoscopy in second-class secondary hospitals.

### Comparison of diagnosis and therapeutic endoscopy in Shanghai and Hunan province

#### Comparison of the application of flexible bronchoscopy in Shanghai in 2002 and 2017

From June 2017 to August 2017, 14 tertiary and secondary hospitals and thoracic and pulmonary hospitals in Shanghai filled out the questionnaire online. The final effective questionnaire covered 11 hospitals, with a valid rate of 78.6%. From February to April 2002, 111 hospitals in Shanghai were surveyed and 52 questionnaires were collected, with a valid response rate of 46.8%.

#### Contrast of Flexible Bronchoscope Ownership

In the two statistical comparisons of flexible bronchoscopes in Shanghai, the average number of flexible bronchoscopes per hospital increased from 2.5 to 17, and the number of electronic bronchoscopes increased from 0.5 to 14.6, an increase of 28.2 times, with a significant increase in the number of flexible bronchoscopes per hospital (Supplementary Table S6).

#### Comparison of the medical staff

In the two surveys conducted in Shanghai, in 2002 the average number of doctors in the respiratory endoscopy centers of each hospital in Shanghai was 5.4, the average number of doctors in tertiary hospitals was 9.4, and the average number of doctors in secondary hospitals was 2.7. In 2017, an average of 10.0 doctors was allocated to the respiratory endoscopy center of each hospital in Shanghai, 12.1 doctors were allocated to tertiary hospitals and 6.0 doctors were allocated to secondary hospitals.

#### Contrast of the launching rate of bronchial interventional diagnosis and therapeutic projects

In 2002, the TBNA development rate of Shanghai Hospital Respiratory Endoscopy Center was 48.1%, stent implantation 21.2%, high frequency electrocoagulation 17.3%, laser 3.8%, freezing 1.9%. Over 2017, the development rate of various diagnostic and therapeutic projects increased in varying degrees (P < 0.05, Supplementary Table S7).

#### Application comparison of flexible bronchoscopy in Hunan province in 2005 and 2017

A total of 15 tertiary and secondary hospitals chest and lung specialized hospitals in Hunan province filled in the network questionnaire. The survey period was from June 2017 to August 2017, and the final effective questionnaire covered 12 hospitals, with the effective rate of 80.0%. From September to December, 2005, Zhou et al. 2 investigated 74 hospitals in Hunan province, collected 53 questionnaires, and the response rate was 71.6%.

#### Comparison of flexible bronchoscopy ownership

In the statistical comparison of the number of flexible bronchoscopy in Hunan province, in 2005, the average respiratory endoscopy center in Hunan province had 0.3 electronic bronchoscopy and 1.2 fiberoptic bronchoscopy. In 2017, every hospital had 5.2 electronic bronchoscopes and 1.4 fiberoptic bronchoscopes on average. It increased by 16.33 and 0.17 times respectively.

#### Comparison of the medical staff

In 2005, each hospital in Hunan province was equipped with an average of 3.2 doctors, 4.5 doctors in tertiary hospitals and 2.0 doctors in secondary hospitals. In 2017, the average number of doctors in respiratory endoscopy centers in each hospital in Hunan province increased by 0.66 times. This is in contrast to the launching rate of bronchial interventional diagnosis and therapeutic projects.

In 2017, the TBNA rate of respiratory endoscopy center of Hunan hospital was 66.7%, 66.7% stent implantation, 75.0% high-frequency electrocoagulation, 8.3% laser, and 33.3% freezing. Compared with 2005, the development rate of each diagnosis and therapeutic project was improved to different degrees (P<0.05, Supplementary Table S8).

#### The application of respiratory endoscopy in Shanghai and Hunan province in 2017

The survey was conducted from June 2017 to August 2017. A total of 14 hospitals in Shanghai and 15 hospitals in Hunan province filled in the online questionnaire, and the final effective questionnaire was conducted in 11 hospitals in Shanghai and 12 hospitals in Hunan province, with the effective rate of 78.6% and 80.0% respectively.

#### Comparison of equipment ownership

According to the survey, the average respiratory endoscopy center in Shanghai has 14.6 electronic bronchoscopes, 2.4 fiberoptic bronchoscopes, 1.7 fluorescent bronchoscopes and 1.2 rigid bronchoscopes. The average hospital in Hunan province has 5.2 electronic bronchoscopes, 1.4 fiberoptic bronchoscopes, 0.8 fluorescent bronchoscopes and 0.3 rigid bronchoscopes. The average number of flexible bronchoscopes increased by 5.8 times in Shanghai from 2002 to 2017, and 3.4 times in Hunan province from 2005 to 2017.

#### Comparison of medical staffs for endoscopy

According to the statistical results, in 2017, Shanghai hospital respiratory endoscopy center was equipped with an average of 10.0 doctors and 6.6 anesthesiologists, among which 83.6% were doctors with master's degree or above. Hospitals in Hunan province have 5.3 doctors and 3.2 anesthesiologists on average, among which 53.4% are doctors with master's degree or above. The average number of doctors in Shanghai increased by 0.85 times from 2002 to 2017 and in Hunan province by 0.66 times from 2005 to 2017.

#### Contrast of the launching rate of bronchial interventional diagnosis and therapeutic projects

In the respiratory endoscopy examination project carried out in Shanghai in 2017, the implementation rate of lung biopsy was 100%, virtual navigation was 63.6%, electromagnetic navigation was 45.5%, and the implementation rate of various technologies in Hunan province was 50.0%, 8.3% and 8.3% respectively. The development rate of the three technologies in Shanghai was significantly higher than that in Hunan province (P<0.05, Supplementary Table S9).

In the comparison of respiratory endoscopic therapeutic projects carried out by Shanghai and Hunan hospitals, the rate of stent implantation was 100%, 66.7%, laser was 63.6%, 8.3%, and lung volume reduction was 63.6%, 16.7%, respectively, with significant differences (P<0.05, Table 7).

## Discussion & Conclusion

Bronchoscopy was initialized in China from 1970s and was earlier initiated in hospitals with higher classes. Routine diagnostic bronchoscopy including BAL and biopsy was performed in nearly all hospitals, while TBNA, thoracoscopy and percutaneous lung biopsy guided by CT were mainly applied in tertiary hospitals. And other newly-developed techniques, such as AFB, NBI, EBUS-GS, VB and ENB, were rarely performed even in tertiary hospitals. Additionally, therapeutic bronchoscopy was likewise performed in those hospitals, which indicated that more experienced doctors majored in bronchoscopy in hospitals with higher classes. This required further development of bronchoscopy in hospitals with lower classes.

Moreover, regarding area distributions, diagnostic and therapeutic bronchoscopy were more commonly performed in Shanghai, Beijing, Shanxi province, Sichuan province and Hunan province, which revealed a multicenter distribution of bronchoscopy in China. This may be beneficial for development of bronchoscopy in surrounding areas.

Though bronchoscopy was now widely used, there were still some problems. One was that the allocated equipment could not meet the increasing needs due to the high incidences of respiratory diseases in China. The other was irrational distributions of bronchoscopy. Another was that the number of allocated doctors specialized in bronchoscopy was small and the skilled ones were few. The results of the study elucidated that only 7.4 doctors and 4.8 anesthetists were allocated for bronchoscopy per hospital. Moreover, most hospitals were lacking in doctors, anesthetists and nurses majoring in bronchoscopy.

It can also be seen that the respiratory endoscopy diagnosis and therapeutic technology in China has developed rapidly in both economically developed and moderately developed regions since the beginning of the 21st century. The phenomenon is attributed to the following two reasons. First of all, the role of respiratory endoscopic intervention in the diagnosis and therapeutic of respiratory diseases is getting higher and higher. Since the beginning of the 21st century, routine respiratory endoscopy diagnosis and therapeutic techniques such as alveolar lavage, TBNA and biopsy forceps biopsy have been carried out in all levels of hospitals. In addition, the development of peripheral lesion ablation, virtual navigation, electromagnetic navigation and microwave therapy and other emerging technologies has made the interventional diagnosis and therapeutic techniques of respiratory endoscopy more and more abundant, and more and more therapeutic methods are available in the face of complex technical problems. Secondly, the industry recognition, policy support and market input. With the gradual deepening of the interventional research of respiratory endoscopy, the routine technology is constantly innovated, the emerging technology is fully developed, and the clinical application guidelines of respiratory endoscopy are also being updated synchronously.

While gains made on all facets of bronchoscopy over the last 30 years in China have been remarkably robust and positive, there are still some main problems to be emphasized: The first one is that here are still major gaps in the configuration of facilities and equipment. In the past 10 years, the diagnosis and therapeutic work of hospitals at all levels has been promoted in an orderly manner, and the bronchoscope diagnosis and therapeutic technology has developed rapidly, but it still cannot meet the needs of patients seeking medical treatment. China is a country with a large population, and the incidence of respiratory diseases has been high in recent years. Although the scale of specialized hospitals is relatively large, general hospitals still account for the majority of regional disease diagnosis and treatment. From the current development of general hospital, hardware problems such as small operation site and few operation rooms still cannot meet the existing clinical needs. Secondly, the resource distribution of bronchial interventional treatment is still unreasonable.

The supply and demand of medical resources are not only affected by the rate of medical work, but also the rational allocation of medical resources. We will establish a new pattern of medical resources distribution in which community hospitals complete routine inspections, large and medium-sized hospitals carry out diagnosis and therapeutic work, and speciality hospitals have their own way to perform diagnosis and therapeutic work. At present, diagnosis and therapeutic technology can only be developed in large hospitals, but the launching rate of emerging technologies, such as electromagnetic navigation, virtual navigation, and microwave diagnosis is still low. While small and medium-sized hospitals can carry out the routine diagnosis and therapeutic bronchial intervention, but poor hardware facilities and small scale, which cannot meet the routine diagnosis work of the majority of community residents, have restricted they from carrying out reasonable triage for patients from large hospitals. In addition, the total number of bronchial endoscopy is still small. At present, there is still a large shortage in the number of respiratory endoscopy physicians in China due to the increasing number of patients in China. Each hospital in China has an average of 7.4 doctors and 4.8 anesthesiologists in the bronchoscopy room. Among them, full-time respiratory endoscopy physicians are even scarcer, and many bronchoscopy rooms are still not equipped with full-time anesthesiologist and anesthesiologist nurses.

Here, we give some corresponding measures and suggestions to the problem. Firstly, the government should pay more attention to the development of bronchoscopy and recognize its importance in the diagnosis and therapeutic work of respiratory diseases. The hardware construction of hospital bronchoscope rooms should be increased, so as to better meet the medical needs of grassroots people. In the second place, implementing the policy of hierarchical diagnosis is the key to improving the utilization rate of medical resources. Community hospitals should improve the rate of routine diagnosis to meet the needs of the masses at the community level. After the initial diagnosis in community hospitals, bronchoscope intervention is carried out by the superior hospitals to alleviate the over-utilization of medical resources in large and medium-sized hospitals. Furthermore, the medical management department should standardize the process of training and examination for endoscopy physicians. It is strictly prohibited for doctors who are not qualified to carry out the diagnosis and therapeutic work of respiratory endoscopy. At the same time, relevant anesthesiologists and nurses in the bronchoscope room should receive corresponding standardized training before taking up their posts, so as to ensure the standardization of medical process.

## Supplementary Material

Supplementary figures and tables.

## Figures and Tables

**Figure 1:**
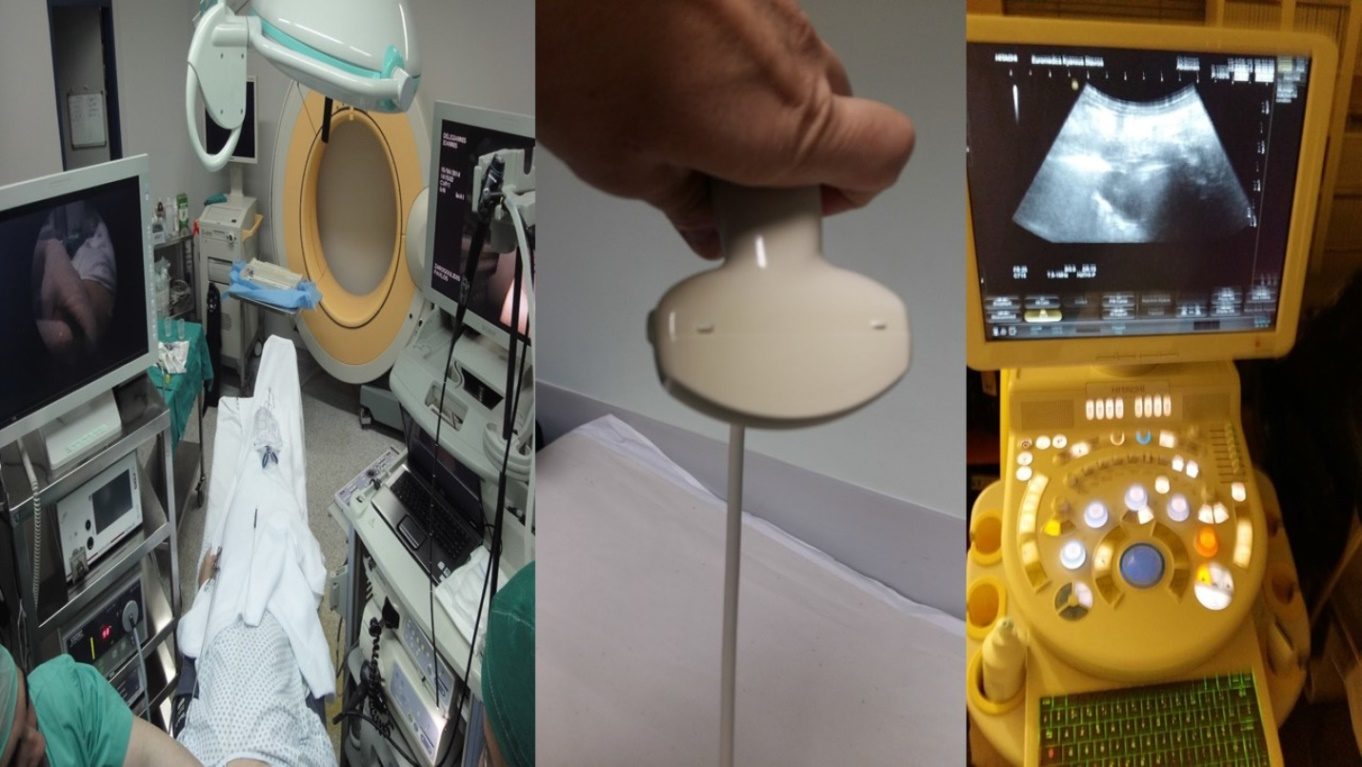
From left to right; Left; O-Arm for navigation, along with rigid and flexible bronchoscope, Middle; convex probe for transthoracic biopsy of superficial thoracic lesions, Right; Ultrasound source for radial, convex probe (EBUS) and transthoracic biopsy.

**Table 1:** The average period of performing diagnostic and therapeutic bronchoscopy

Hospitals	Diagnostic bronchoscopy (years)	Therapeutic bronchoscopy (years)
Overall (n=319)	19.7	7.4
First-class tertiary hospital (n=221)	23.2	8.7
Second-class tertiary hospital (n=38)	15.4	5.3
First-class secondary hospital (n=56)	9.9	3.9
Second-class secondary hospital (n=4)	4.3	1.5

**Table 2:** Diagnostic bronchoscopy performed in hospitals with different classes per month

Hospitals	BAL (n, %)	Biopsy (n, %)	TBNA (n, %)	EBUS-TBNA (n, %)	AFB (n, %)	NBI (n, %)	Thoracoscopy (n, %)
Overall (n=319)	299 (93.7)	317 (99.4)	207 (64.9)	110 (34.5)	84 (26.3)	91 (28.5)	182 (57.1)
First-class tertiary hospital (n=221)	213 (96.4)	221 (100)	169 (76.5)	104 (47.1)	80 (36.2)	80 (36.2)	146 (66.1)
Second-class tertiary hospital (n=38)	36 (94.7)	38 (100)	21 (55.3)	6 (15.8)	3 (7.9)	4 (10.5)	20 (52.6)
First-class secondary hospital (n=56)	46 (82.1)	54 (96.4)	17 (30.4)	0 (0)	1 (1.8)	7 (12.5)	16 (28.6)
Second-class secondary hospital (n=4)	4 (100)	4 (100)	0 (0)	0 (0)	0 (0)	0 (0)	0 (0)

**Table 3:** Diagnostic bronchoscopy performed in hospitals with different classes per month cont.

Hospitals	EBUS-GS (n, %)	Percutaneous lung biopsy guided by CT (n, %)	VB (n, %)	ENB (n, %)
Overall (n=319)	88 (27.6)	211 (66.1)	51 (16.0)	14 (4.4)
First-class tertiary hospital (n=221)	82 (37.1)	160 (72.4)	48 (21.7)	14 (6.3)
Second-class tertiary hospital (n=38)	4 (10.5)	28 (73.7)	3 (7.9)	0 (0)
First-class secondary hospital (n=56)	2 (3.6)	23 (41.1)	0 (0)	0 (0)
Second-class secondary hospital (n=4)	0 (0)	0 (0)	0 (0)	0 (0)

**Table 4:** Therapeutic bronchoscopy performed in hospitals with different classes per month

Hospitals	Electrocautery (n, %)	Laser ablation (n, %)	Balloon dilation (n, %)	APC (n, %)	BLVR (n, %)
Overall (n=319)	209 (65.5)	79 (24.8)	222 (69.6)	168 (52.7)	55 (17.2)
First-class tertiary hospital (n=221)	164 (74.2)	67 (30.3)	173 (78.6)	141 (63.8)	51 (23.1)
Second-class tertiary hospital (n=38)	21 (55.3)	6 (15.8)	20 (52.6)	18 (47.4)	3 (7.9)
First-class secondary hospital (n=56)	21 (37.5)	4 (7.1)	26 (46.4)	8(14.3)	1 (1.8)
Second-class secondary hospital (n=4)	3 (75.0)	2 (50.0)	3 (75.0)	1 (25.0)	0 (0)
**Hospitals**	**Bronchoplasty (n, %)**	**Stent implantation (n, %)**	**RFA (n, %)**	**Radioactive seeds implantation (n, %)**	**Microwave therapy (n, %)**
Overall (n=319)	60 (18.8)	198 (62.1)	49 (15.4)	57 ( 17.9)	68 ( 21.3)
First-class tertiary hospital (n=221)	57 (25.8)	159 (71.9)	41 (18.6)	43 ( 19.5)	53 ( 24.0)
Second-class tertiary hospital (n=38)	3 (7.9)	17 (44.7)	5 (13.2)	7 ( 18.4)	10 ( 26.3)
First-class secondary hospital (n=56)	0 (0)	20 (35.7)	3 ( 5.4)	7 ( 12.5)	5 ( 8.9)
Second-class secondary hospital (n=4)	0 (0)	2 (50.0)	0 (0)	0 (0)	0 (0)

**Table 5:** The average cases receiving diagnostic and therapeutic bronchoscopy in each hospital per month

Hospitals	Diagnostic bronchoscopy (n)	Therapeutic bronchoscopy (n)
Overall (n=319)	155.2	28.4
First-class tertiary hospital (n=221)	201.0	35.3
Second-class tertiary hospital (n=38)	79.4	18.1
First-class secondary hospital (n=56)	35.3	3.7
Second-class secondary hospital (n=4)	20.0	2.3

**Table 6:** The average number of equipment per hospital

Hospitals	Electronic bronchoscope (n)	Fiberoptic bronchoscope (n)	Rigid bronchoscope (n)	Auto-fluorescence bronchoscope (n)	EBUS (n)	Cryotherapy (n)
Overall (n=319)	4.4	1.7	0.3	0.4	0.5	0.8
First-class tertiary hospital (n=221)	5.5	2.1	0.4	0.5	0.6	0.9
Second-class tertiary hospital (n=38)	2.9	1.2	0.1	0.1	0.2	0.7
First-class secondary hospital (n=56)	1.4	0.9	0	0	0	0.2
Second-class secondary hospital (n=4)	1.8	0.5	0.3	0	0	0.5
**Hospitals**	**Electrocautery (n)**	**APC(n)**	**Laser ablation(n)**	**Microwave(n)**	**Flexible thoracoscope (n)**	**Rigid thoracoscope (n)**
Overall (n=319)	0.7	0.9	0.2	0.2	0.6	0.3
First-class tertiary hospital (n=221)	0.9	0.7	0.3	0.2	0.7	0.4
Second-class tertiary hospital (n=38)	0.6	0.6	0.2	0.2	0.4	0.2
First-class secondary hospital (n=56)	0.3	1.6	0	0.1	0.2	0
Second-class secondary hospital (n=4)	0.5	0.3	0	0	0	0.5

**Table 7:** Respiratory endoscopic therapeutic technology in Shanghai and Hunan province in 2017 (N/%)

Area	Stent implantation	Balloon Dilatation	Electrocoagulation	Laser	BLVR	APC	Cryotherapy	Bronplasty	Microwave	Radioactive seeds implantation
Shanghai (n=11)	11 (100)	10 (90.9)	9 (81.8)	7 (63.6)	7 (63.6)	6 (54.5)	5 (45.5)	5 (45.5)	4 (36.4)	2 (18.2)
Hunan (n=12)	8 (66.7)	10 (83.3)	9 (75.0)	1 (8.3)	2 (16.7)	7 (58.3)	4 (33.3)	2 (16.7)	4 (33.3)	1 (8.3)
χ^2^	4.439	0.007	0.012	5.492	5.316	0.057	0.028	1.093	0.082	0.007
P	0.035	0.936	0.912	0.019	0.021	0.812	0.867	0.296	0.775	0.936
