# Supplementary material for: The development of bronchoscopy in China: a national cross-sectional study
Source: J Cancer. 2020 Jul 20;11(19):5547–55. doi: 10.7150/jca.47183 (PMC7477436; doi:10.7150/jca.47183)
Supplement: Supplementary file 1 — Supplementary figures and tables. [file jcav11p5547s1.pdf]

Supplementary Table 1. Diagnostic endoscopy in teaching and non-teaching hospitals（%）

Hospital	BAL	Biopsy	TBNA	NBI	TBNA	EBUS-TBNA	Thoracoscopy	TBI	AFB	VB	ENB
Teaching hospital（n=113）	112 (99.1)	113 (100)	88 (77.9)	46 (40.7)	86 (76.1)	67 (59.3)	86 (76.1)	57 (50.4)	52 (46.0)	31 (27.4)	9 (8.0)
Non-teaching hospital（n=206）	187 (90.8)	204 (99.0)	119 (57.8)	45 (21.8)	125 (60.7)	43 (20.9)	96 (46.6)	31 (15.0)	32 (15.5)	20 (9.7)	5 (2.4)
χ^2	8.634	0.096	12.084	12.735	7.755	47.673	25.925	45.761	34.956	17.069	5.332
P	0.003	0.757	<0.001	<0.001	0.005	<0.001	<0.001	<0.001	<0.001	<0.001	0.021

Supplementary Table 3. Therapeutic endoscopy in teaching and non-teaching hospitals （%）

Hospital	Electrocautery	Laser	Cryotherapy	Balloon dilation	APC	Bronchoplasty	Thermoplasty	Stent implantation	Radioactive seeds implantation	Microwave
Teaching hospital（n=113）	87 (77.0)	39 (34.5)	64 (56.6)	92 (81.4)	77 (68.1)	33 (29.2)	41 (36.3)	89 (78.8)	23 (20.4)	29 (25.7)
Non-teaching hospital（n=206）	122 (59.2)	40 (19.4)	86 (41.7)	130 (63.1)	91 (44.2)	22 (10.7)	19 (9.2)	109 (52.9)	34 (16.5)	39 (18.9)
χ^2	10.197	8.925	6.494	11.560	16.814	17.548	34.989	20.709	0.737	1.972
P	0.001	0.003	0.011	0.001	<0.001	<0.001	<0.001	<0.001	0.391	0.160

Supplementary Table 4. The average area and number of respiratory endoscopic center per hospital

Hospitals	The average area (m^2)	The average number (n)
**Overall (n=319)**	122.7	2.2
**First-class tertiary hospital (n=221)**	150.3	2.4
**Second-class tertiary hospital (n=38)**	99.7	2.2
**First-class secondary hospital (n=56)**	36.4	1.5
**Second-class secondary hospital (n=4)**	25.0	1.5

Supplementary Table 5. The average number of flexible bronchoscope in each hospital per month in different areas of China

Areas	Electronic bronchoscope (n)	Fiberoptic bronchoscope (n)	Flexible bronchoscope (n)	*Overall population (million)	#Average number per million population

**Shanghai**	14.6	2.4	17.0	24.20	0.7025
**Beijing**	10.9	1.9	12.8	21.73	0.5851
**Tianjin**	7.8	1.0	8.8	15.62	0.5655
**Hainan province**	1.2	0.7	1.9	9.17	0.3272
**Chongqing**	5.9	1.9	7.8	30.48	0.2543
**Ningxia Hui Autonomous Region**	1.2	0.5	1.7	6.75	0.2519
**Shaanxi province**	5.9	3.6	9.5	38.13	0.2473
**Jinlin province**	4.9	1.4	6.3	27.33	0.2300
**Qinghai province**	1.0	0.3	1.3	5.93	0.2192
**Fujian province**	3.7	2.8	6.5	38.74	0.1668
**Shanxi province**	3.8	2.3	6.1	36.82	0.163
**Heilongjiang province**	4.0	2.0	6.0	37.99	0.1579

**Guizhou province**	3.4	1.7	5.1	35.55	0.1447
**Gansu province**	3.3	0.5	3.8	26.10	0.1437
**Hubei province**	5.0	2.9	7.9	58.85	0.1344
**Zhejiang province**	5.5	1.8	7.3	55.9	0.1288
**Xinjiang Uygur Autonomous Region**	2.0	1.0	3.0	23.98	0.1251
**Inner Mongolia Autonomous Region**	1.5	1.2	2.7	25.20	0.1058
**Hunan province**	5.2	1.4	6.6	68.22	0.0965
**Yunnan province**	3.1	1.1	4.2	47.71	0.0871
**Jiangsu province**	5.6	1.4	7.0	79.99	0.0868

**Sichuan province**	5.8	1.1	6.9	82.62	0.0838
**Henan province**	3.7	4.2	7.9	95.32	0.0822
**Guangxi Zhuang Autonomous Region**	2.1	1.5	3.6	48.38	0.0747
**Liaoning province**	2.3	0.8	3.1	43.78	0.0704
**Anhui province**	3.0	1.1	4.1	61.96	0.0658
**Hebei province**	3.1	1.7	4.8	74.70	0.065
**Shandong province**	4.7	1.6	6.3	99.47	0.0637
**Jiangxi province**	2.0	0.7	2.7	45.92	0.0583
**Guangdong province**	3.4	2.8	6.2	109.99	0.0565

Flexible bronchoscope included electronic and fiberoptic bronchoscope.

*The population was from National Bureau of Statistics of China in 2016.

#The average number refers to flexible bronchoscope.

Supplementary Table 6. Comparison of equipment ownership in each hospital in Shanghai between 2002 and 2017

Time(year)	Flexible bronchoscope	electronic bronchoscope	Fiberoptic bronchoscope
**2002**	2.5	0.5	2.0
**2017**	17	14.6	2.4

Supplementary Table 7. Comparison of the launching rate of bronchial diagnosis and therapeutic projects in hospitals located in Shanghai in 2002 and 2017

Time	Cytology	Biopsy	BAL	TBNA	Stent implantation	Electrocoagulation	Laser	Cryotherapy
**2002**	100	98.1	94.2	48.1	21.2	17.3	3.8	1.9

**2017**	100	100	100	81.8	100	81.8	63.6	45.5
χ^2	-	0.747	0.001	4.161	21.489	15.489	21.849	15.235
**P**	-	0.388	0.970	0.041	<0.001	<0.001	<0.001	<0.001

Supplementary Table 8. Comparison of the launching rates of respiratory endoscopic diagnosis and therapeutic projects in Hunan hospitals in 2005 and 2017

Time	cytology	Biopsy	BAL	TBNA	Stent implantation	Electrocoagulation	Laser	Cryotherapy
**2005**	100	79.2	69.8	3.8	18.9	32.1	0	3.8
**2017**	100	100	100	66.7	66.7	75.0	8.3	33.3
χ^2	-	1.703	3.316	25.096	8.905	5.830	4.486	6.981
**P**	-	0.192	0.069	<0.001	0.003	0.016	0.034	0.008

Supplementary Table 9. Respiratory endoscopic technology in Shanghai and Hunan province in 2017 (%)

Area	Biopsy	BAL	lung biopsy	Thoraco scopy	TBNA	EBUS-TBNA	NBI	Fluoros copy	VB	NBI	ENB
Shanghai （n=11）	11(100)	11(100)	11(100)	10(90.9)	9(81.8)	7(63.6)	7 (63.6)	7 (63.6)	7 (63.6)	6 (54.5)	5 (45.5)
Hunan （n=12）	12 (100)	12 (100)	6(50.0)	9(75.0)	8 (66.7)	4(33.3)	4(33.3)	5(41.7)	1(8.3)	6(50.0)	1(8.3)
χ^2	-	-	5.074	0.207	0.123	1.072	1.072	0.404	5.492	0.040	2.402
P	-	-	0.024	0.649	0.725	0.300	0.300	0.525	0.019	0.842	0.121
